# Advanced age is not a barrier to chronic intracortical single-unit recording in rat cortex

**DOI:** 10.3389/fnins.2024.1389556

**Published:** 2024-05-15

**Authors:** Nicholas F. Nolta, Michael B. Christensen, Patrick A. Tresco

**Affiliations:** ^1^Department of Biomedical Engineering, University of Utah, Salt Lake City, UT, United States; ^2^Division of Urology, Department of Surgery, University of Utah School of Medicine, Salt Lake City, UT, United States; ^3^Department of Otolaryngology – Head & Neck Surgery, University of Utah School of Medicine, Salt Lake City, UT, United States

**Keywords:** microelectrode recording arrays, foreign body response, astrogliosis, aging, biocompatibility

## Abstract

**Introduction:**

Available evidence suggests that as we age, our brain and immune system undergo changes that increase our susceptibility to injury, inflammation, and neurodegeneration. Since a significant portion of the potential patients treated with a microelectrode-based implant may be older, it is important to understand the recording performance of such devices in an aged population.

**Methods:**

We studied the chronic recording performance and the foreign body response (FBR) to a clinically used microelectrode array implanted in the cortex of 18-month-old Sprague Dawley rats.

**Results and discussion:**

To the best of our knowledge, this is the first preclinical study of its type in the older mammalian brain. Here, we show that single-unit recording performance was initially robust then gradually declined over a 12-week period, similar to what has been previously reported using younger adult rats and in clinical trials. In addition, we show that FBR biomarker distribution was similar to what has been previously described for younger adult rats implanted with multi-shank recording arrays in the motor cortex. Using a quantitative immunohistochemcal approach, we observed that the extent of astrogliosis and tissue loss near the recording zone was inversely related to recording performance. A comparison of recording performance with a younger cohort supports the notion that aging, in and of itself, is not a limiting factor for the clinical use of penetrating microelectrode recording arrays for the treatment of certain CNS disorders.

## Introduction

Paralysis currently affects 1 in 50 Americans, 16% of which are completely unable to move any part of their body (Spinal Cord Injury Zone, 2009). In addition, limb loss affects 1 in 190 Americans (Ziegler-Graham et al., 2008). In both populations, the loss of motor function imposes a significant lifelong burden. Implantable microelectrode recording arrays are a type of implantable biomedical device being developed to restore function for such patients. Penetrating microelectrode arrays implanted in the human cortex, along with additional computational equipment, have provided paralyzed patients with intentional control over computer cursors (Hochberg et al., 2006; Simeral et al., 2011) and robotic devices (Hochberg et al., 2012; Collinger et al., 2013). More recently, studies have shown that it is possible for individuals with traumatic high cervical spinal cord injury (SCI) to perform coordinated reaching and grasping movements using their own paralyzed arm and hand muscles, which are reanimated through functional electrical stimulation and controlled using volitional cortical signals through a chronically implanted intracortical recording array (Ajiboye et al., 2017).

The potential group of patients with paralysis resulting from traumatic injury or stroke that may be treated with such devices includes a significant number of older adults, 56% of which are over the age of 50, and 33.5% of which are over the age of 60 (Spinal Cord Injury Zone, 2009). In addition, 80% of individuals with limb loss are over 45, while 42% are over 65 (Ziegler-Graham et al., 2008). Moreover, most young patients will eventually reach at least middle age. For instance, in a case of high tetraplegia due to spinal cord injury in a 20-year-old patient, the life expectancy may reach 57 years (Spinal Cord Injury Zone, 2009).

During aging, the immune system undergoes changes including: a reduced number of naïve B and T cells, a decrease in stimulated phagocytosis and reactive oxygen species (ROS) production by neutrophils, and dysregulation of immune cell signaling pathways (Weiskopf et al., 2009; Ponnappan and Ponnappan, 2011). These changes result in prolonged wound healing (Gosain and DiPietro, 2004) and increased levels of proinflammatory cytokine production in cases of neuroinflammation (Weiskopf et al., 2009; Ponnappan and Ponnappan, 2011).

The brain also changes with age. Microglia, the brain’s resident macrophage, acquire a more reactive phenotype (von Bernhardi et al., 2010), become larger and less ramified, express increased markers of activation, and produce higher levels of proinflammatory cytokines such as tumor necrosis factor alpha (TNF-alpha), interleukin 1-beta (IL-1β) and interleukin 6 (IL-6) (Perry et al., 1993; Ye and Johnson, 1999; Sierra et al., 2007; Hart et al., 2012; Ritzel et al., 2015). In addition, astrocytes become more numerous and hypertrophic (Amenta et al., 1998). Neuronal loss and a reduction in the number of dendrites and synapses also occurs (Juraska and Lowry, 2012), rendering the aged mammalian brain more susceptible to such neurodegenerative diseases as Alzheimer’s (Lindsay et al., 2002) and Parkinson’s (Van Den Eeden et al., 2003). Therefore, the brain of older patients may present a uniquely challenging environment for the successful implementation of neuroprosthetic control that uses penetrating multishaft, microelectrode arrays chronically implanted into the surface of the brain. The multiple injuries associated with the implantation of a penetrating multishaft microelectrode array and the accompanying neuroinflammation associated with the chronic foreign body response (FBR), may play a more significant role in the aged mammalian brain and thus reduce chronic recording performance compared to such implants in younger individuals.

Clinical studies indicate that older patients have poorer clinical outcomes following ischemic stroke (Macciocchi et al., 1998), traumatic brain injury (Hukkelhoven et al., 2003), and aneurysmal subarachnoid hemorrhage (Lanzino et al., 1996). Similarly, older rats show increased loss of neural tissue following experimental injuries (Rosen et al., 2005; Kumar et al., 2013). A recent study using a single planar penetrating array in the rat cortex reported a significantly reduced FBR and improved recording performance over a six-week period in 4-week-old rats compared to a slightly older group (9 weeks at implantation) (Sharon et al., 2023). Moreover, clinical studies using deep brain stimulation (DBS) with devices implanted in older patients show increased rates of complications (Voges et al., 2007), decreased clinical benefit (Saint-Cyr et al., 2000; Charles et al., 2002; Welter et al., 2002), and increased rates of cognitive and behavioral impairment (Saint-Cyr et al., 2000).

**Figure 1:**
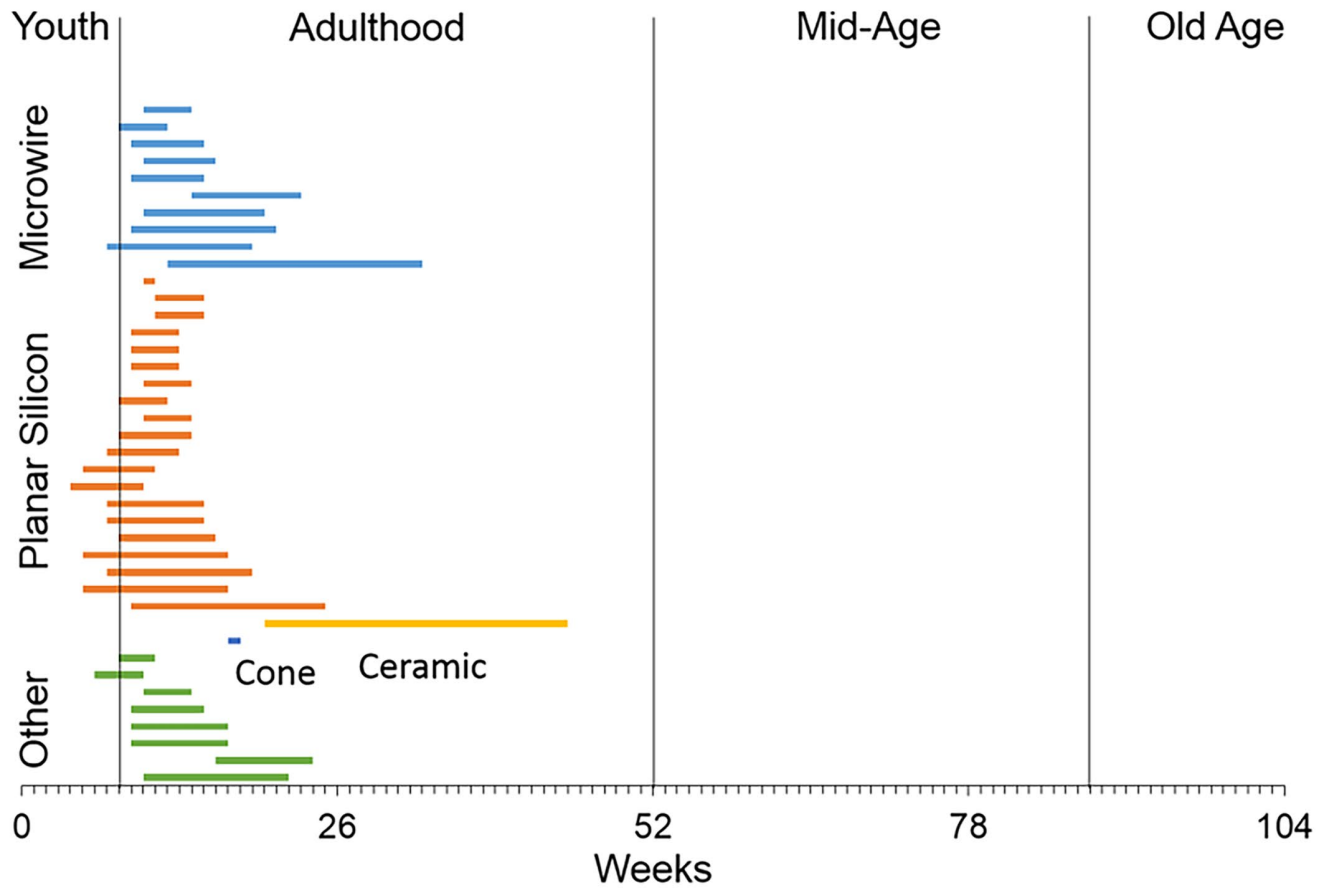
A literature review of the ages of rats used in studies that have employed different types of recording microelectrode arrays implanted into the rat brain. The majority of studies used male rats in early adulthood or 8–16 weeks of age (Levine et al., 1963; Kennedy, 1989; Turner et al., 1999; Venkatachalam et al., 1999; Shain et al., 2003; Kim et al., 2004; Biran et al., 2005; Spataro et al., 2005; He et al., 2006; Biran et al., 2007; McConnell et al., 2007; Rennaker et al., 2007; Seymour and Kipke, 2007; Stice et al., 2007; Zhong and Bellamkonda, 2007; Eriksson Linsmeier et al., 2009; Hascup et al., 2009; Lu et al., 2009; McConnell et al., 2009; Ward et al., 2009; Azemi et al., 2010; Hirshler et al., 2010; Lind et al., 2010; Nelson et al., 2010; Winslow et al., 2010; Winslow and Tresco, 2010; Andrei et al., 2011; Azemi et al., 2011; Freire et al., 2011; Harris et al., 2011; Lewitus et al., 2011; Skousen et al., 2011; Thelin et al., 2011; Welkenhuysen et al., 2011; Woolley et al., 2011; Potter et al., 2012; Prasad et al., 2012; Rao et al., 2012).

To the best of our knowledge, no studies have examined whether penetrating, multishaft chronic recording arrays can function chronically after implantation in the brain of older animals as a model for their use in older patients. A survey of the literature indicates that most of the studies using rats to study recording performance of central nervous system penetrating recording microelectrodes have used males that are in the early part of adulthood (Figure 1), while a significant number of patients enrolled in clinical BCI research studies have been much older, ranging in age from 52 to 66 (Hochberg et al., 2006; Simeral et al., 2011; Hochberg et al., 2012; Collinger et al., 2013; Ajiboye et al., 2017). To address this issue, we implanted a 4×4 microelectrode recording array into the 18-month old rat motor cortex, analyzed the recording performance in the unanesthetized condition over a 3-month indwelling period, compared the recording performance to a younger cohort that received the same implant over the same indwelling period, and assessed the foreign body response (FBR) at the study endpoint using an immunohistochemical approach.

## Materials and methods

### Microelectrode arrays

**Figure 2:**
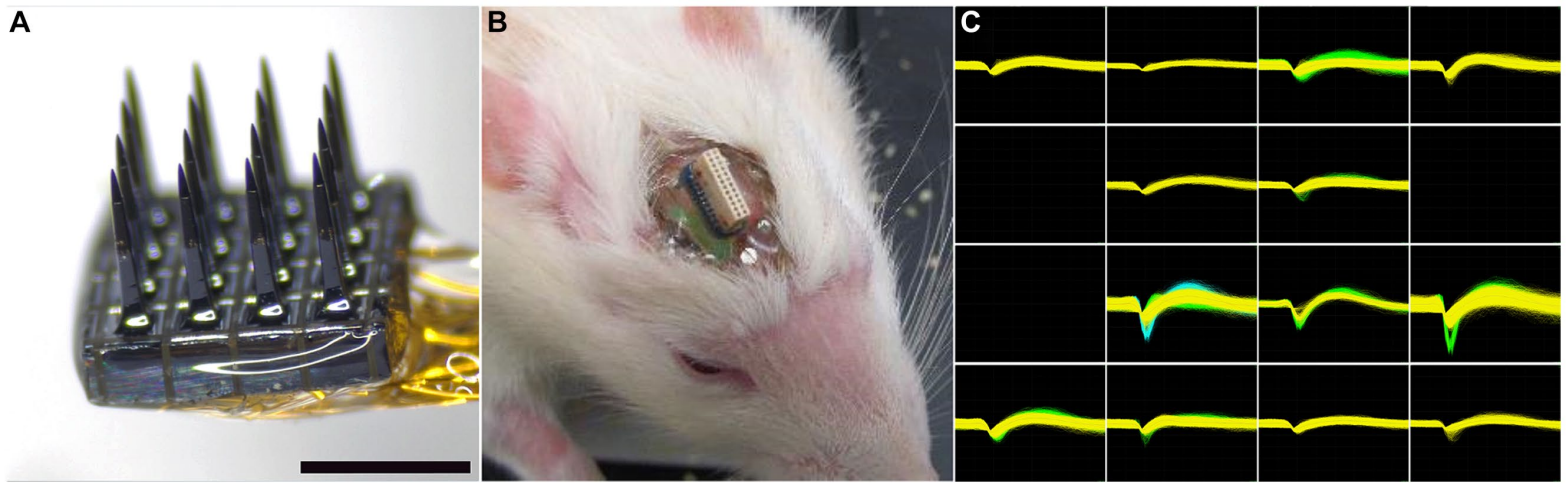
**(A)** Representative 4×4 UEA before implantation. Scale bar = 1 mm. **(B)** Representative headstage on a 19-month-old rat 4 weeks after implantation. The photocurable acrylic polymer was clear and allowed visualization of the screws and the underlying cortex. **(C)** An example of waveforms from an 18-month-old rat 4 weeks after implantation, showing 21 units on 13 individual microelectrodes. All units are on the same scale, with the y-axis maximum set at 700 μV and the x-axis spanning 1,600 μs.

The microelectrode arrays used in this study were purchased from Blackrock Microsystems (Salt Lake City, UT). The array, referred to as the Utah Electrode Array (UEA) had a 4×4 rectangular grid of 1 mm long microelectrode shafts spaced 400 μm apart (Figure 2A) and was similar in overall design to the 10×10 microelectrode recording arrays used in several nonhuman primate studies (Santhanam et al., 2006; Barrese et al., 2013) and several clinical studies (Hochberg et al., 2006; Simeral et al., 2011; Hochberg et al., 2012; Collinger et al., 2013; Ajiboye et al., 2017). The wiring diagram relating connector pins to the locations of each microelectrode recording tip in each array was supplied by the manufacturer to allow correlation of end-point histology with recording performance analysis. Each UEA was cleaned in an agitated solution of 1% Alconox, followed by rinsing in sterile distilled water (DI) water (3x), acetone, isopropanol, and then sterile DI water (3x). The cleaned arrays were then packaged for ethylene oxide (EtO) sterilization at the University of Utah Hospital Surgical Processing Center and allowed to outgas for a minimum of 48 h prior to implantation.

### Animal surgery

All procedures involving animals were approved by the University of Utah Animal Care and Use Committee. Male Sprague Dawley rats were purchased at approximately 8 months of age and then housed in the University of Utah vivarium in pairs until they achieved 18 months of age before implantation. Eight animals were implanted with one 4×4 UEA each in the right hemisphere of the motor cortex as described below.

Each rat was anesthetized with 5% isoflurane/oxygen and its head was shaved. Each animal was then positioned in a stereotactic frame and its scalp disinfected with 70% isopropyl alcohol followed by betadine (repeated three times). A midline incision was made along the length of the skull and the skull was exposed with sterile cotton-tipped applicators. The skull was then treated with 2% hydrogen peroxide and dried with a sterile cotton-tipped applicator. Four stainless steel screws (Fine Science Tools, Foster City, CA) were manually turned into pilot holes angled into the temporal ridge that were predrilled with a pneumatic dental drill. A rectangular craniotomy, approximately 5×5 mm, was created over the right primary motor cortex using a pneumatic dental drill. Sterile PBS rinses were used to remove bone debris and minimize heating during the drilling procedure. After the bone plug was removed, the dura was pierced and reflected to the side of the craniotomy with a hooked 25-gauge needle. A sterilized UEA was implanted stereotactically with the aid of a 0.25 mm stainless steel rod (Small Parts, Miami, FL) attached to the back side of the device with acrylic adhesive which was held in the stereotaxic manipulator and positioned over the center of the craniotomy. Using the stereotaxic manipulator, the UEA was slowly inserted into the motor cortex until the base of the UEA reached the top of the cortical surface, as visualized by the surgeon using a stereomicroscope. Pneumatic insertion was not employed in this study at the recommendation of the array manufacturer. The reference wire was inserted into the adjacent cortex using forceps. Uncontrolled bleeding was not observed during any of the implantation procedures. The area between the craniotomy edge and the implanted UEA was filled with sterile Kwik-Sil (World Precision Instruments, Sarasota, FL). The ground wire was then wrapped around a bone screw and then tunneled a short distance under the skin behind the head incision. After the Kwik-Sil set (5 min), the stainless steel rod was carefully cut as close to the UEA base as possible with a wire cutter. Then the UEA and its omnetic connector were secured between the four bone screws using a photocurable acrylic adhesive (1187-M, Dymax, Torrington, CT) applied in a series of layers. Figure 2B shows a close-up of the head stage in a 19-month-old rat 4 weeks after implantation.

### Electrophysiological recordings

The rats were allowed to recover for 1 week, after which spontaneous single-unit recordings were obtained from unanesthetized, freely moving animals for a period of 5 min twice a week, as previously described (Nolta et al., 2015; Black et al., 2018), to simulate the recording of volitional movements that occur in clinical studies. Recordings were collected using a Cerebus data acquisition system (Blackrock Microsystems, Salt Lake City, UT) and analyzed offline using Plexon Offline Sorter (Plexon, Dallas, TX) (Figure 2C). Single-unit action potentials were isolated in principal component space using a manually assisted sorting algorithm. Signal-to-noise ratio (SNR) was determined by dividing the peak-to-peak amplitude of the average waveform of an isolated unit by the RMS noise floor of the microelectrode. The recording performance for a particular week was determined by observing the highest number of units recorded from a particular electrode during that week, or the highest SNR observed for a given unit during the week.

### Failure analysis

In order to track the cause and time course of device related failures, animals were examined at each recording session for signs of head stage loosening, which was termed hardware failure. Due to the age of the animals, the occurrence of natural death was an issue and was referred to as natural causes. If the loss of recordable single-unit action potentials could not be explained by either of these two cases they were called FBR related. The majority of animals used for recording performance made it to the study endpoint and were sacrificed 12 weeks after implantation, at 21 months of age.

### Euthanasia and tissue preparation

Twelve weeks after implantation, the animals were anesthetized with 5% isoflurane and perfused transcardially with 200 mL of phosphate buffered saline pH 7.4 (PBS) followed by 200 mL of 4% paraformaldehyde in PBS. The brains and the arrays were carefully removed from the skull. Brains and arrays were post-fixed for 24 h in 4% paraformaldehyde in PBS. Whole brains were equilibrated in a 30% sucrose solution in PBS until they no longer floated in the storage container (2–3 days), which was stored under refrigeration. The sucrose treated brains were then cut in 30 μm sections in the horizontal plane using a cryostat at −22 degrees Celsius.

### Immunohistochemical methods

Free-floating brain sections were incubated overnight in blocking solution consisting of PBS with 4% v/v goat serum (Invitrogen, Carlsbad, CA), 0.5% v/v Triton-X 100, and 0.1% sodium azide. Selected sections were then incubated individually overnight with one of the following primary antibodies in blocking solution: CD68 (ED-1, AbD Serotec, Raleigh, NC, 0.25 μg/mL) to identify activated macrophages and microglia, IBA-1 (Wako Chemicals USA, Inc., Richmond, VA, 0.5 μg/mL) to label all macrophages and microglia, IgG (biotinylated goat anti-rat IgG, Southern Biotec, 0.5 μg/mL) to assess BBB leakage, GFAP (DAKO North America Inc., Carpinteria, CA, 2.9 μg/mL) to examine astrocyte cytoskeleton location and hypertrophy, NeuN (EMD Millipore, Billerica, MA, 1 μg/mL) to identify neuronal nuclei, and NF160 (Sigma-Aldrich, St. Louis, MO, 5 μg/mL) to visualize axons and dendrites. Sections were then rinsed three times with PBS for 3 h each before being incubated overnight with appropriate fluorescently-labeled secondary antibodies plus 10 μM DAPI to label cell nuclei. Sections were rinsed again three times with PBS for 3 h each at room temperature on a rocker. The same protocol was used on explanted arrays to identify adherent cell types. Sections were mounted on slides and cover slipped in Fluoromount-G (Southern Biotec), then imaged. For retrieved arrays, images were taken using a confocal microscope with a 5x air objective or a 40x water objective with the array submerged in PBS in a petri dish.

### Measurement of cavity volume

To calculate the volume of damaged neural tissue at the implantation site, referred to as the cavity volume, 2D cavity areas were imaged in horizontal sections that were devoid of NeuN, GFAP, or neurofilament immunoreactivity and manually outlined in Photoshop (Adobe Systems Inc., San Jose, CA). The prismoidal formula:


$$V=\frac{L}{3}\left(A+\sqrt{\mathrm{AB}}+B\right)$$

was then used to estimate the cavity volume V lying between two sequential sections separated by distance L and having 2D void areas A and B, then summed over all sections.

### Image quantification

Immunofluorescence was quantified inside a circular 100 μm radius centered in each microelectrode track near the microelectrode tip. The immunofluorescence was compared to the average immunofluorescence in a similar region of cortex on the contralateral hemisphere in each section, which served as the control image. Areas devoid of normal DAPI-stained tissue were excluded from average intensity measurements. Since each microelectrode shaft was spaced at 400 μm intervals, there was no overlap of images.

### Comparison with a younger cohort

We compared electrophysiological results, as well as failure analysis data, with the results of our previous study using a younger cohort of rats which was studied by the same team of investigators using the same recording and analytical methods (Nolta et al., 2015).

### Statistics

To better understand the relationship between recording performance and the FBR, we compared the relative fluorescence from individual microelectrode tip recording zones that recorded at least one single-unit action potential in the last recording session to the microelectrode tip recording zones that did not record any action potential but were otherwise functional, using a Student’s *t*-test. Comparisons between old and young cohorts were also conducted using a Student’s *t*-test. Correlations between recording performance and time post-implantation also were determined using regression analysis. *T*-tests were performed using Microsoft Excel with and without unequal variances where applicable. *p*-values below 0.05 were considered significant. All data is represented as mean ± SEM.

## Results

### Rat model

Twelve male Sprague Dawley rats were purchased at 8 months of age and housed in the University of Utah vivarium with free access to food and water until they reached 18 months of age. At the time of implantation, they had an average weight of 935 ± 48 g (for comparison, the young animals in our previous cohort (Nolta et al., 2015) had an average starting weight of 334 ± 11 g, *p* < 0.0001). Four rats died of natural causes during the aging period prior to the beginning of the study and were not included in the analysis.

### Failure analysis

**Figure 3:**
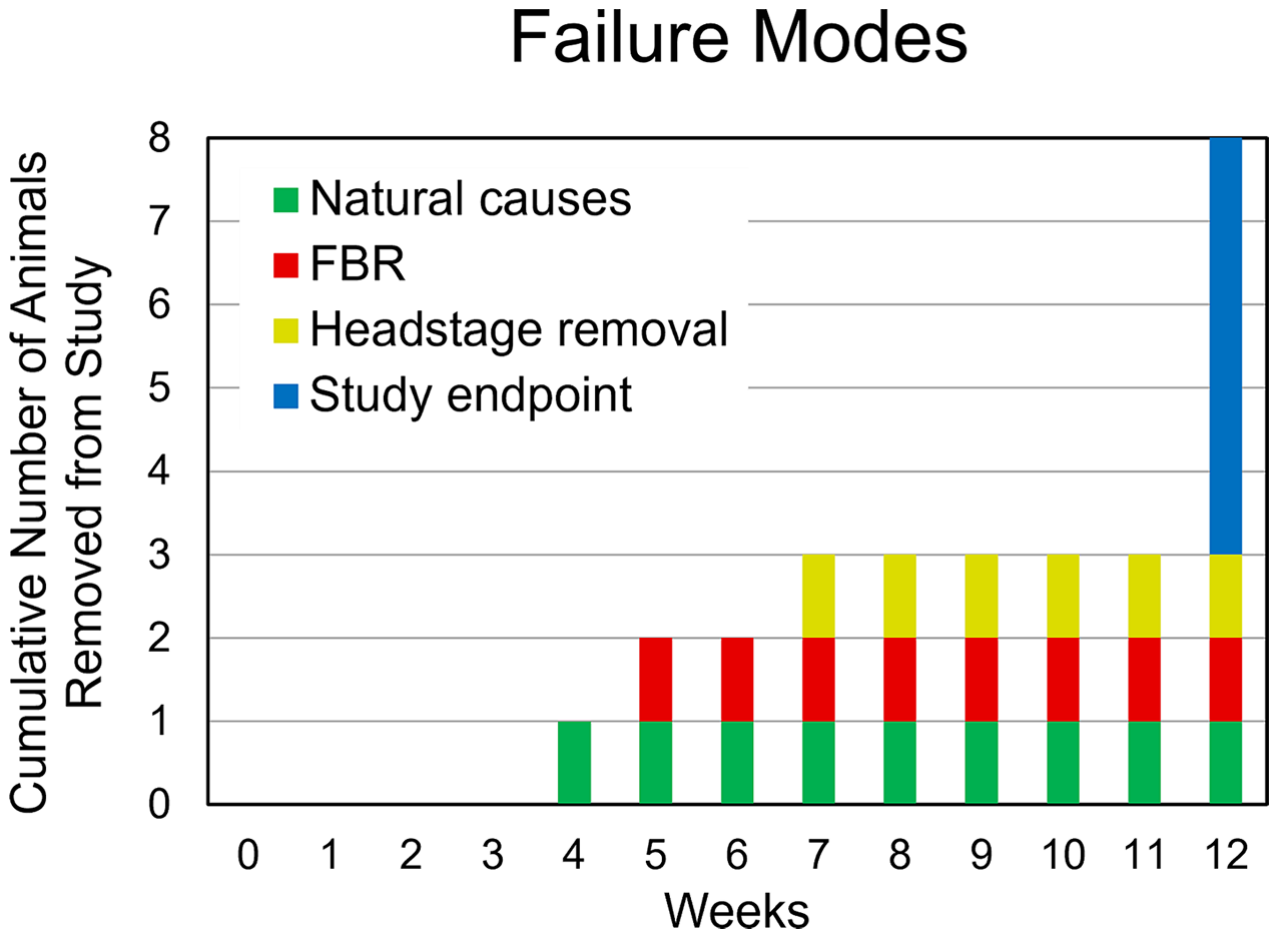
Summary of failure modes. One animal died of natural causes after 4 weeks (green). Another animal had a complete loss of recording at 4 weeks despite a functional, well anchored UEA. It was found to have had a large WBC-filled cavity in the cortex at the site of implantation (red). Another animal was removed due to headstage loosening (yellow). The remaining animals (*N* = 5) continued to yield action potentials throughout the 12-week study (blue).

One animal died of complications 4 weeks after implantation. Another animal experienced complete loss of single-unit recordings 5 weeks after implantation and was lethargic. After consultation with the veterinary staff that animal was euthanized. Upon perfusion and dissection, this animal was found to have a large stroke–like, white blood cell filled cavity in the superficial cortex under the array. Another animal’s headstage loosened and was found in the animal’s cage 7 weeks after implantation. The remaining animals (*N* = 5) yielded single-unit action potential recordings through the 12-week indwelling period (Figure 3). The mean skull thickness and SEM taken from the 21-month-old rats at the end of the study measured at the midline of the parietal bone near bregma was 1,114 ± 101 μm (*N* = 5). Our previous younger male cohort of the same rat strain implanted with the same array by the same team of investigators using similar methods (Nolta et al., 2015) was used to compare modes of failure with the older rats in this study (Table 1). The results showed that no animals in the younger cohort (*N* = 28) died of natural causes. There was a significantly greater number of younger rats that lost headstages due to early hardware failure and there was a significantly higher percentage of older rats that made it to the study end point (Table 1).

**Table 1:** Comparison of failure modes between young and older rats that were implanted with a 4×4 UEA.

Failure mode	Young animals	Older animals	*p*-value
Natural causes	0/28 (0%)	1/8 (13%)	0.06
Headstage (hardware)	15/28 (54%)	1/8 (13%)	0.04
FBR	11/28 (39%)	1/8 (13%)	0.17
End point	2/28 (7%)	5/8 (63%)	<0.001

Data for the young cohort was derived from a previously published study by the same group (Nolta et al., 2015).

### Electrophysiology

**Figure 4:**
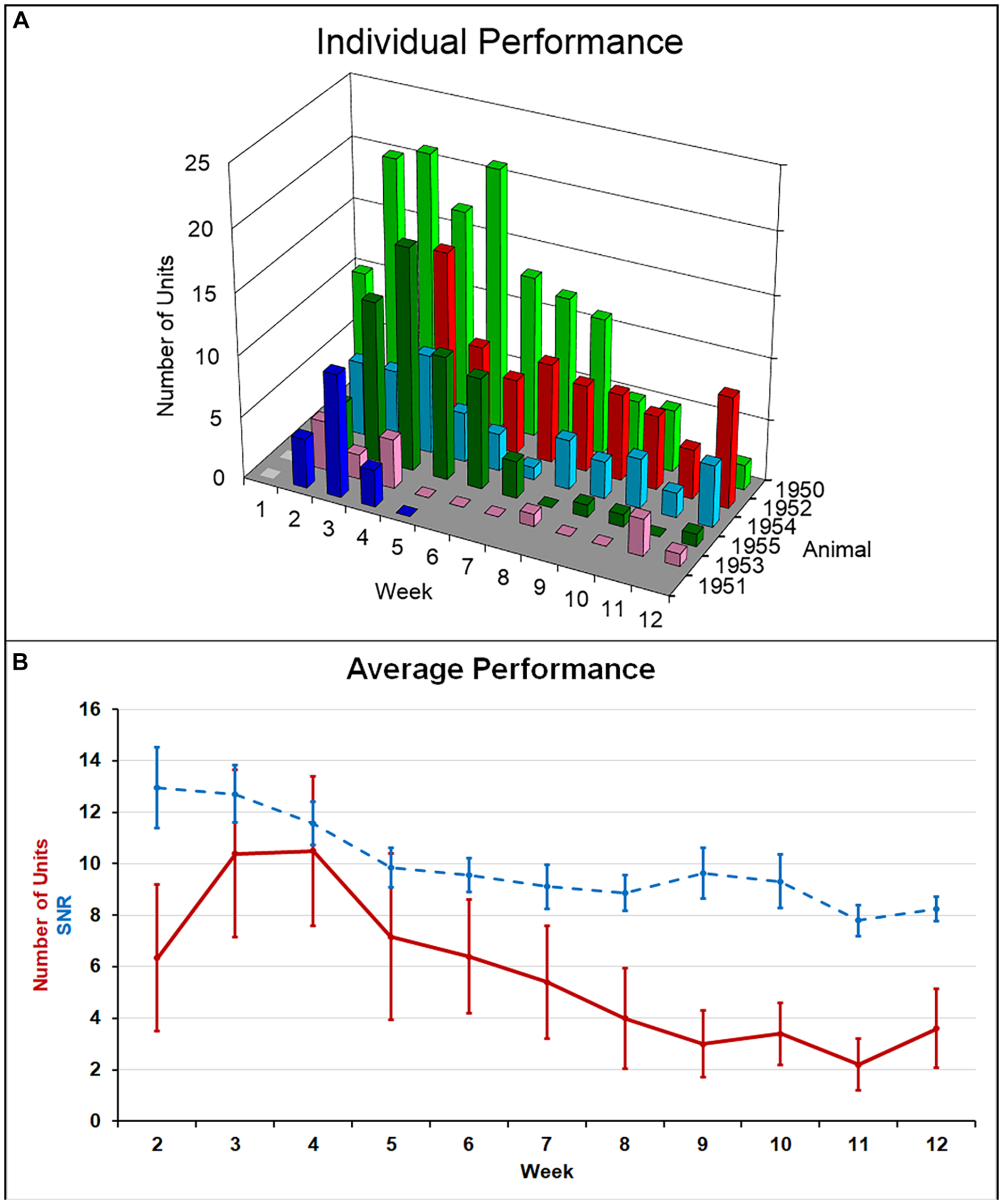
**(A)** Summary of recording performance over time and across animals. **(B)** The average number of single units recorded per animal (solid line) was highest at weeks 3–4 and decreased to a lower level thereafter. Average SNR across all units (dashed line) slowly decreased over the indwelling period. Both of these negative correlations with time were significant (*p* < 0.002).

Single-unit action potentials were detected in the majority of animals during the recording sessions. The animal that died of natural causes and the animal who lost its headstage never produced single-unit recordings. Recording performance from the remaining animals (*N* = 6) varied across animals and across time, but was generally highest 3–4 weeks after implantation and then gradually decreased to a lower level over the 12-week indwelling period (Figure 4). Recording performance for individual microelectrode shafts varied over the 12-week period and only three individual microelectrode shafts in three separate animals recorded action potentials every session over the entire 12-week period. Both the average number of units and the SNR were inversely correlated with time (*p* < 0.002) (Figure 4B). A comparison of the number of electrodes that recorded at least one single-unit action potential during a given week over the total number of possible electrodes that could have recorded that week for the older cohort compared to our previous younger cohort is presented in Table 2. At no time point during the 12-week recording period did the older cohort yield a lower percentage of possible electrodes that recorded a single-unit action potential than the younger group.

**Table 2:** Number of electrodes that recorded at least one single-unit action potential during a given week over the total number of possible electrodes that could have recorded during that week (percentage).

Time	Young animals	Older animals	*p*-value
Week 2	39/128 (30%)	30/96 (31%)	0.90
Week 3	35/144 (24%)	35/80 (44%)	0.003
Week 4	30/144 (21%)	46/96 (48%)	<0.001
Week 5	13/48 (27%)	33/96 (34%)	0.38
Week 6	5/31 (16%)	25/80 (31%)	0.11
Week 7	0/31 (0%)	21/80 (26%)	0.001
Week 8	1/31 (3%)	17/80 (21%)	0.02
Week 9	2/31 (6%)	12/79 (15%)	0.22
Week 10	0/31 (0%)	14/79 (18%)	0.01
Week 11	3/31 (10%)	11/79 (14%)	0.55
Week 12	2/31 (6%)	17/78 (22%)	0.06

Data for young animals was derived from a previously published study by the same group (Nolta et al., 2015).

### Explanted arrays

**Figure 5:**
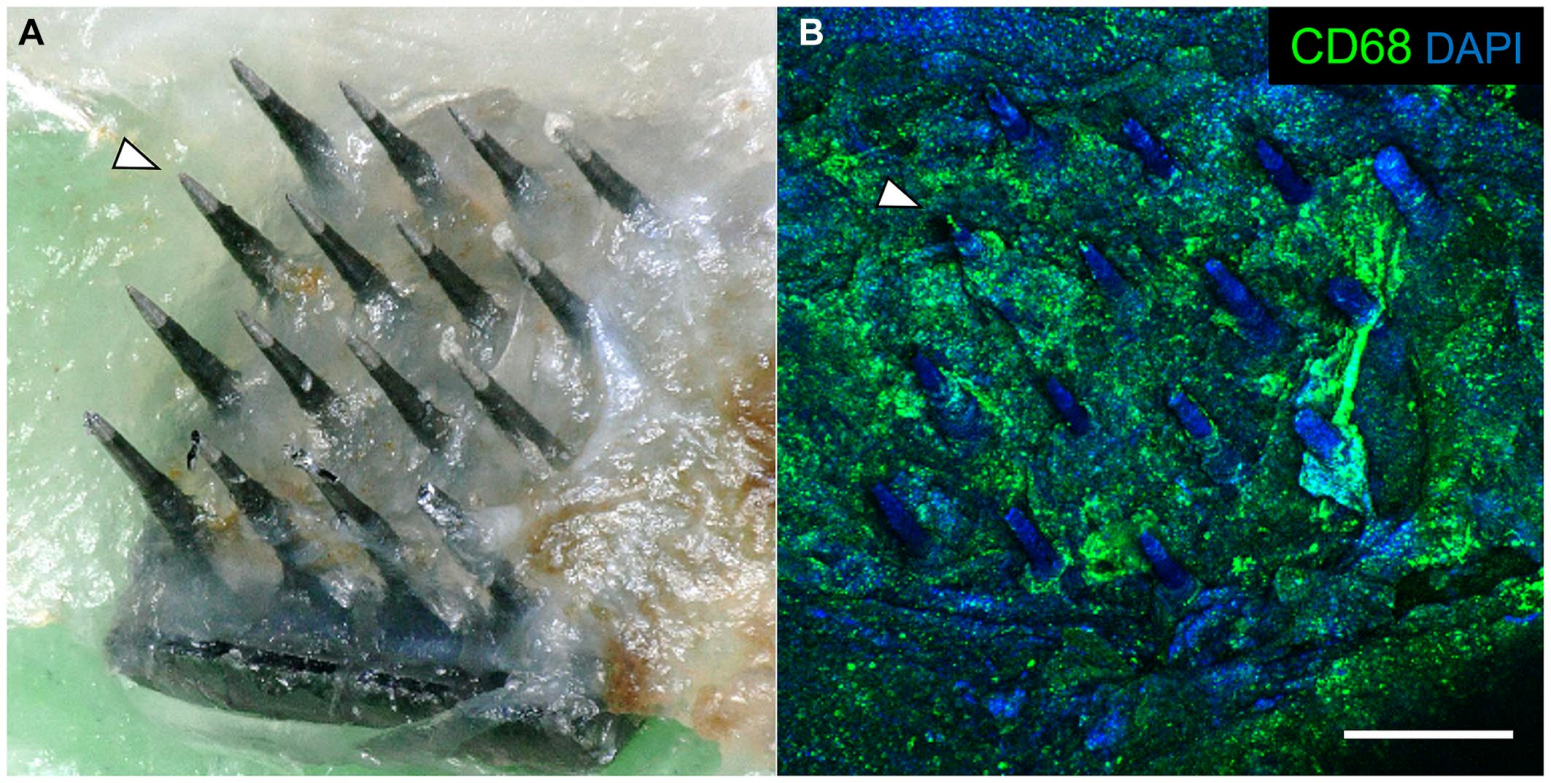
**(A)** Photograph of a retrieved UEA 12 weeks after implantation in a 21-month-old rat motor cortex. The base was encapsulated in connective tissue. Arrowhead indicates the same microelectrode shaft identified in both panels. **(B)** Same array as in panel A, showing immunoreactivity against CD68 (green) and DAPI (blue) associated with the encapsulation tissue. Scale bar = 500 μm.

After careful removal of the headstage from the skull bone, we observed that each array was surrounded by skull bone that appeared to have regenerated from the edge of the original craniotomy to surround the base of the array. Each array was in the same orientation as they were originally implanted. The arrays were easily removed from the fixed brain. Subsequent analyses showed that the underside of each explanted array was encapsulated in fibrotic tissue (Figure 5A). The amount of encapsulation tissue ranged from covering the base of the array and the upper one third of the microelectrode shafts (as shown in Figure 5A), to a more significant reaction that enveloped most of the microelectrode shaft length. Immunohistochemical analyses of explanted arrays revealed that the encapsulation tissue was positive for collagen I, negative for both NeuN and neurofilament, and contained little GFAP immunoreactivity. In addition, it contained a large amount of CD68+ immunoreactivity, indicating that activated macrophages were present in the adherent tissue, as well as other unidentified cells that were DAPI positive but did not react with the antibodies tested (Figure 5B).

### Description of the FBR

**Figure 6:**
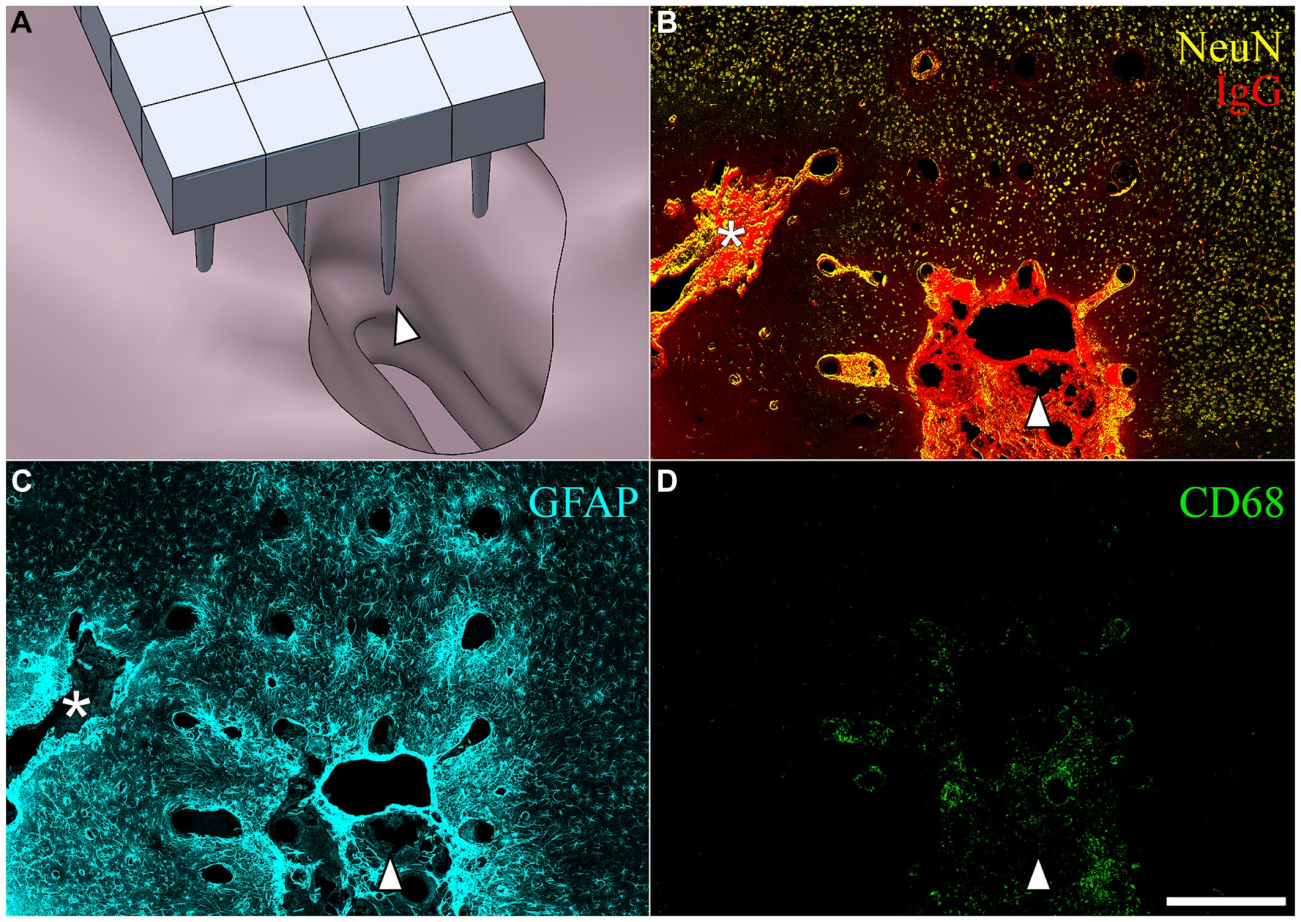
**(A)** SolidWorks rendering of a lesion cavity in relation to an implanted UEA after a 3-month indwelling period in a 21-month-old rat cortex. **(B–D)** Representative horizontal sections from the same animal shown in **(A)** but with the orientation of the array shifted slightly to show an area of deep cortex near the tips of individual microelectrode shafts. The white arrowhead in each panel is provided for viewer orientation with the image shown in the CAD drawing **(A)**. **(B)** Shows BBB leakage (IgG immunoreactivity in red) and neuronal nuclei (NeuN immunoreactivity in yellow) at a depth near the tips of the array. Another, more narrow lesion cavity not visible in **(A)** can be seen extending off the edge of the array footprint indicated with the white asterisk. IgG was highly concentrated within the lesion cavity and perilesion tissue, but was minimally present in intact parenchyma. **(C)** Hypertrophic astrocytes (GFAP) were visible around microelectrode tracks and in the perilesion cavity. **(D)** CD68 was observed in the lesion and perilesion zone but was not as dominant near the tips of microelectrode tracks. Scale bar = 500 microns.

After removing the array from the cortical surface, a cavity was visible on the surface of the brain where the microelectrode was located. These varied in size. Figure 6A shows a computer-generated reconstruction produced from serial horizontal sections of one such cavity which extended beyond the base of the array. In horizontal sections, the cavities were identified as regions devoid of NeuN, GFAP, or neurofilament immunoreactivity, as well as normal DAPI positive cortical tissue (Figures 6B–D). The border of the cavity had higher immunoreactivity for IgG, decreased immunoreactivity for neuronal nuclei and neurofilament, and increased immunoreactivty for GFAP. The cavities were variable in size (Table 3), but tended to be pyramidal in shape, becoming narrower with depth into the cortex. The appearance of the perfused neural tissue at the margins of the cavity had a slightly darker color than the rest of the perfused brain. In the majority of cases, the superficial aspect of the cavity near the cortical surface appeared to encompass most of the base of the array. In a few cases, cavities narrowed in sections closer to the microelectrode tips. The cavities in some horizontal sections were filled with loose connective tissue that contained CD68+ immunoreactivity and were immunoreactive for IgG (Figures 6B,D). The average cavity volume estimated from serial horizontal sections was 2.5 ± 1.2 mm^3^.

**Table 3:** Summary of recording performance, lesion volume, and connective tissue coverage of explanted arrays for the five animals that showed single-unit recording on at least one microelectrode shaft through the study end point.

Animal	Electrodes with units at week 12	Lesion volume (mm^3^)	Tissue on explanted array
1954	5	0.85	Underside of base
1952	8	0.86	Underside of base
1953	1	1.21	Underside of base
1950	2	2.22	Entire base of array
1955	1	7.35	Entire base of array

Higher recording performance was associated with smaller lesion volumes and less encapsulation tissue.

### Correlation of recording performance and end-point histology

**Figure 7:**
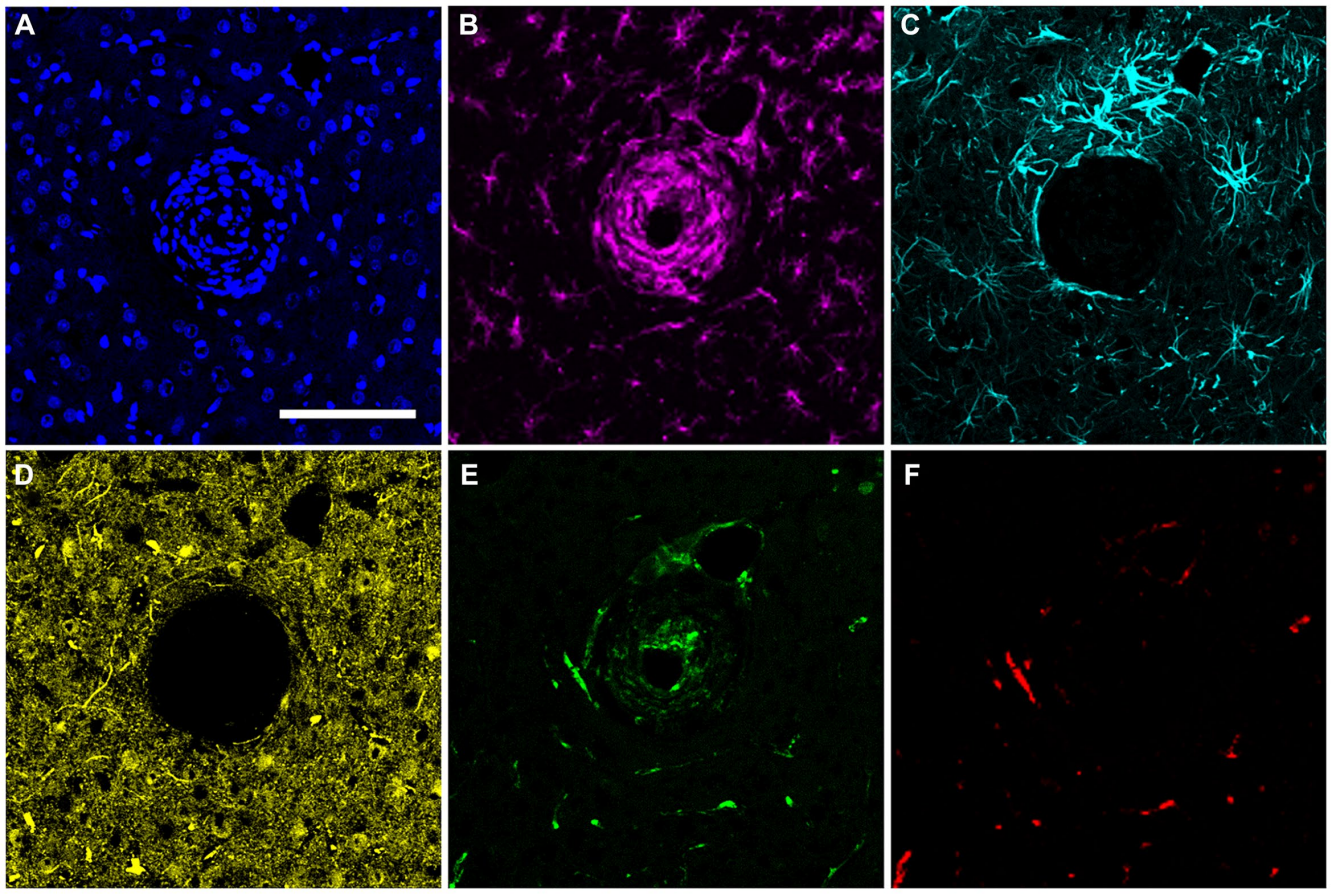
Representative high-magnification view of the foreign body response in horizontal sections taken at a depth near the tip of a microelectrode shaft 12 weeks after implantation. **(A)** DAPI positive nucleus of each cell. **(B)** IBA1 immuno-reactive cells. **(C)** GFAP immunoreactivity. **(D)** NeuN and NF160 immunoreactivity. **(E)** CD68 immunoreactivity. **(F)** Immunoreactivity for IgG. Scale bar = 100 μm.

**Figure 8:**
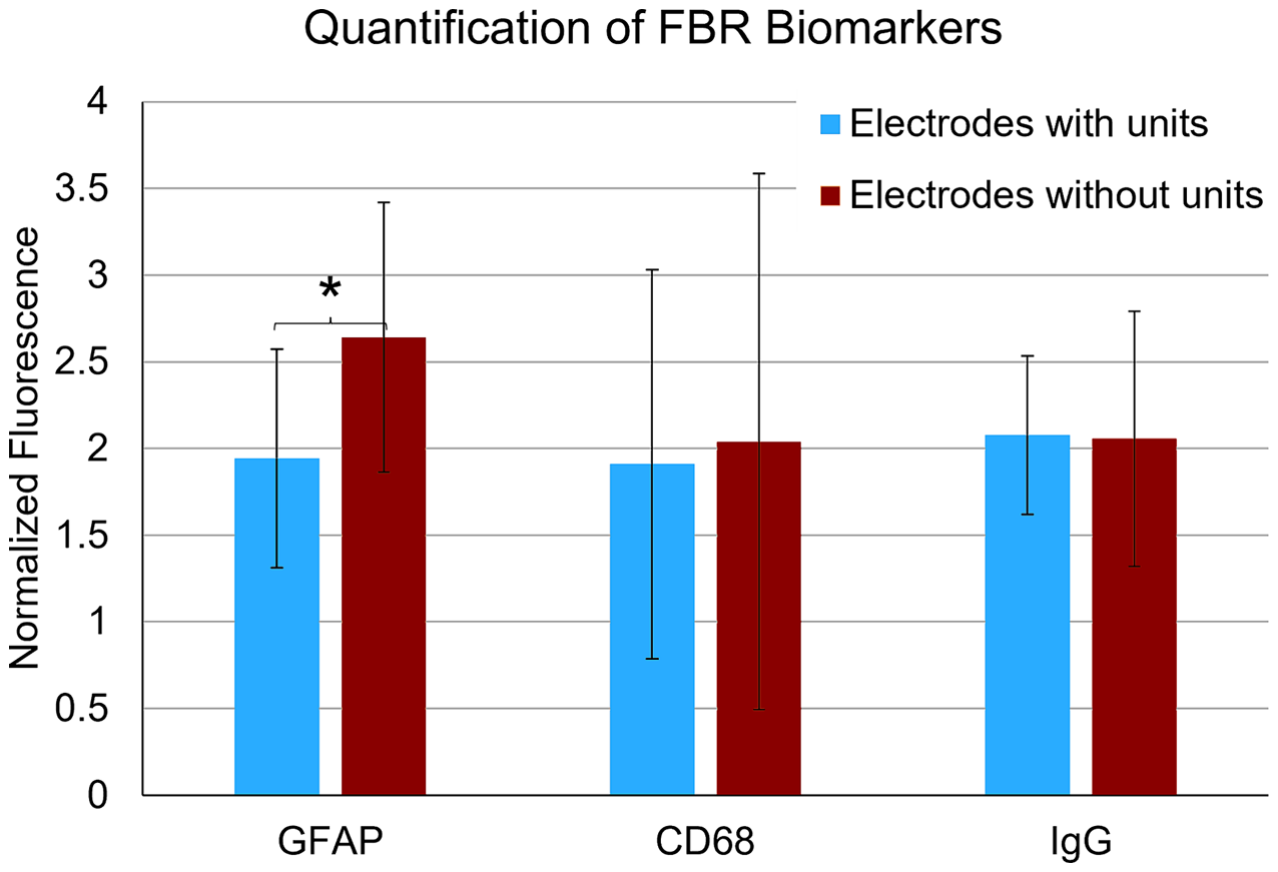
Quantification of FBR biomarkers in brain tissue within a 100 μm radius of the tip of individual microelectrodes. Microelectrodes that recorded at least one unit in the final week of recording had significantly lower levels of astrocyte cytoskeleton (GFAP) (*p* = 0.047). Levels of activated macrophages/microglia (CD68) and IgG were not statistically different. *p < 0.05.

In the final week of recording, all surviving animals had arrays that recorded from at least one microelectrode. Arrays with the smallest cavity volume recorded a larger number of single units than those with larger lesion cavities (Table 3). The two arrays with the largest cavities and with the largest amount of encapsulation tissue on the explanted array had 1 or 2 microelectrodes that were recording single units at the 12-week time point. These were located away from the cavity. Immunohistochemical analysis of FBR-associated biomarkers in brain tissue at the level of the microelectrode tips is shown in Figure 7. The microelectrode shaft was surrounded by cells that were positive for IBA1 and, to a lesser extent, CD68, indicating that the most proximal layer of cells was either activated microglia or blood derived activated macrophages. These cells were uniformly distributed around the microelectrode shaft. This area was devoid of other biomarkers of the FBR, as well as biomarkers of neuronal cell bodies and their processes, indicated by a lack of NeuN and NF160 immunoreactivity (Figure 7D). GFAP immunoreactivity was also observed in this zone (Figure 7C), but it rarely incorporated the IBA1+ cells. IgG immunoreactivity in this area was minimal (Figure 7F). A quantitative analyses (Figure 8) indicated that GFAP was significantly higher near the microelectrode tips that did not record units as compared to those that did (*p* = 0.047), while levels of CD68 and IgG immunoreactivity were similarly distributed whether microelectrodes recorded at least one unit or not.

## Discussion

In this study, we implanted 18-month-old male rats with a 4×4 UEA into the motor cortex and then examined single-unit recording performance in the implanted, freely moving animals at weekly intervals over a 12-week indwelling period. We observed single-unit action potentials over the entire indwelling period. Single-unit recording performance was most robust in the month following implantation, and then gradually declined over the subsequent 2-month period. These findings are similar to what has been reported using the same recording array in younger adult rats (Nolta et al., 2015; Black et al., 2018; Cody et al., 2018). More importantly, when we compared the single-unit recording yield of the older cohort in this study to a younger male cohort (3 months old) of the same rat strain over a 12-week period, the recording performance yield was as good as, and in some weeks significantly better than, the younger cohort (Table 2).

To the best of our knowledge, this is the first study that has examined chronic recording performance and the FBR in older rats using a penetrating recording array of any type. Based on a 25-month median lifespan for male Sprague Dawley rats (Cameron et al., 1982) and a 79-year median lifespan for American males, an approximate equivalent human age for the rats used in this study was approximately 53 years at implantation and 62 years at the study endpoint (Sengupta, 2013). This age group is similar to the patient population that has been studied in clinical trials that have evaluated the UEA as a treatment of paralysis (Hochberg et al., 2006; Simeral et al., 2011; Hochberg et al., 2012; Collinger et al., 2013; Ajiboye et al., 2017).

The chronic recording performance and aspects of the FBR observed here were somewhat unexpected given the body of literature that suggests that the effects of aging might have had a negative impact on recording performance given the older rat’s increased sensitivity to ischemic injury and neuroinflammation (Lanzino et al., 1996; Macciocchi et al., 1998; Saint-Cyr et al., 2000; Charles et al., 2002; Welter et al., 2002; Hukkelhoven et al., 2003; Rosen et al., 2005; Voges et al., 2007; Kumar et al., 2013). We found that the FBR of the older rats at the study endpoint was similar to that previously described using the same array implanted in younger rat motor cortex (Nolta et al., 2015; Black et al., 2018). Moreover, the FBR around individual microelectrode shafts near the recording tips showed a stereotypical response, as previously described in studies performed in younger rats, irrespective of the indwelling period or implanted microelectrode type (Collias and Manuelidis, 1957; Biran et al., 2005, 2007; Rennaker et al., 2007; McConnell et al., 2009; Winslow et al., 2010; Winslow and Tresco, 2010; Skousen et al., 2011; Potter et al., 2012; Prasad et al., 2014).

With regard to FBR biomarkers, we observed immunoreactivity for IgG and CD68 surrounding the UEA after a 3-month indwelling period, which also was reported using younger animals (Nolta et al., 2015; Black et al., 2018). In this study, neither biomarker correlated with recording success at the study endpoint and did not appear significant near the recording tips. Several groups have reported that the distribution of intraparenchymal IgG due to BBB leakiness decreases with time after implantation for single-shank planar silicon microelectrode arrays in the cortex of young rats (Potter et al., 2012) and in mice (Ravikumar et al., 2014). Several studies using younger cohorts of rats have reported that CD68 immunoreactivity is also reduced at longer indwelling periods compared to shorter periods (McConnell et al., 2009; Potter et al., 2012; Prasad et al., 2014; Ravikumar et al., 2014).

We observed a significant amount of connective tissue underneath the base and on the upper parts of the microelectrode shafts of the retrieved arrays. This observation also was reported in cortically implanted UEAs in younger rats (Nolta et al., 2015; Black et al., 2018; Cody et al., 2018) and in UEAs explanted from non-human primates after long indwelling periods (Barrese et al., 2013). Connective tissue was present on all explanted arrays in the older cohort. The connective tissue was NeuN and neurofilament negative and contained little GFAP, indicating that it was non-neural tissue and likely of meningeal origin. The amount of connective tissue was highly variable between animals and appeared to correlate closely with the volume of the lesion cavity. We speculate that brain tissue lost due to vascular and tissue damage that accompany device implantation caused by the multiple, closely spaced penetrating shafts is eventually filled in with non-neural, collagenous tissue. This interpretation agrees with several studies that showed that a stab wound injury performed with the 4×4 UEA resulted in a stroke-like lesion cavity in younger rats, and focal hemorrhage near and below UEAs implanted acutely and then removed in the cortex of human patients (House et al., 2006; Waziri et al., 2009; Nolta et al., 2015). In a recent study, key genes that mediate the acute injury and neuroinflammatory response, along with genes critical to the function of the BBB, were not significantly different between a UEA implanted group and a UEA stab wound group using the same implantation approach (Bennett et al., 2018). That study showed that the effect of UEA insertion-related trauma dominates early wound healing surrounding the implant.

We observed that single-unit recording performance in the older rats was inversely related to the lesion cavity size. Lesion cavities, similar to those observed here, have also been produced in laser induced experimental stroke models that ablate a single descending arteriole or ascending venule (Shih et al., 2013), as well as in both young implanted and stab-wounded rats using a 4×4 UEA (Nolta et al., 2015; Black et al., 2018). Together, these observations suggest that neural tissue loss observed after UEA implantation in rats is likely related to the amount of vascular damage caused by device implantation, and not necessarily the result of the persistent inflammation that accompanies the chronic phases of the FBR (Nolta et al., 2015; Bennett et al., 2018; Black et al., 2018; Cody et al., 2018). In general, the lesion cavity in the older cohort examined here was similar to that reported previously using younger rats of the same strain using the same array and implantation approach (Nolta et al., 2015).

Lesion or stroke-like cavities have only been reported in rats in a subset of studies that used multi-shank microelectrode arrays in younger rats (Williams et al., 2007; Ward et al., 2009; Saxena et al., 2013; Black et al., 2018; Sharon et al., 2023), and not in those using simple planar single-shank silicon microelectrode arrays or with a few planar microelectrode shafts. Williams et al. found significantly altered impedance spectra for microwires associated with lesions, but did record single units (Williams et al., 2007). Several studies have shown lesion cavities in their published work but did not specifically describe their occurrence (Ward et al., 2009; Saxena et al., 2013; Black et al., 2018). Two studies by Prasad et al. did not analyze neural tissue loss, but did report increased injury during implantation, as evidenced by bleeding that was associated with reduced recording performance (Prasad et al., 2012, 2014). Given the high vasculature density of the rat cortex, vasculature damage resulting from insertion of a UEA with its closely spaced multiple silicon microelectrode shafts seems inevitable, irrespective of the age of the rat used.

Unlike CD68 and IgG immunoreactivity, we observed that single-unit recording performance was inversely related to the level of GFAP immunoreactivity near microelectrode recording tips. We found that microelectrodes that did not record any units in the final week of recordings had significantly higher levels of GFAP immunoreactivity within a 100 μm radius of the recording tip (the presumptive recording zone). These findings agree with a previous report that used an implanted 4×4 UEA in younger rats in the same target tissue, in which higher levels of GFAP corresponded with reduced SNR for individual microelectrodes within the same array (Nolta et al., 2015). This relationship may be causal if hypertrophic astrocytes displace neuronal soma from the recording zone, or if GFAP immunoreactivity is a good indicator of persistent neuroinflammatory stimuli which may silence neuronal activity through any number of other mechanisms including demyelination.

Our results suggest that functional issues related to anchorage of the array may be underappreciated. Fibrotic tissue buildup has been proposed to cause movement of multi-shaft recording arrays following implantation injury for free floating arrays in nonhuman primates (Barrese et al., 2013) and cats (Rousche and Normann, 1998; Maynard et al., 2000; McCreery et al., 2010). In a retrospective analysis of numerous experiments with UEAs chronically implanted in macaques, the authors determined that 53% of all slowly-progressing recording failures were due to fibrotic tissue buildup that dramatically changed the orientation of the arrays from their original implantation position (Barrese et al., 2013). We observed very little movement of the arrays in the aged rat cohort examined in this study, which may have been due to their thicker skulls compared to younger rats. At the time of array retrieval, the skull bone at the craniotomy site was consistently measured to be over a millimeter in thickness. While we can only speculate, it appeared that the edges of the craniotomy surrounding the array regenerated somewhat during the 12-week indwelling period and where very close to the base of the UEA at explant, which may have helped prevent headstage movement.

These findings have implications for studies in larger animals and humans. For one, they support the idea that reducing the impact of vascular damage during implantation and the magnitude of the persistent FBR during the indwelling period will likely improve chronic single-unit recording performance. Previous studies suggest that this may be accomplished through a number of device design changes including reducing device surface area (Seymour and Kipke, 2007; Skousen et al., 2011), increasing device permeability (Skousen et al., 2014), reducing device size (Kozai et al., 2012), reducing device stiffness (Harris et al., 2011), increasing spacing between microelectrode shafts (McConnell et al., 2007, 2009), administering antiflammatory drugs locally (Zhong and Bellamkonda, 2007) or systemically (Rennaker et al., 2007; Potter-Baker et al., 2015), and minimizing vascular damage during insertion (Kozai et al., 2010). It is likely that a combination of such approaches will be needed to improve chronic recording performance and improve device biocompatibility. The results of this study are also encouraging for the future of microelectrode–based therapeutic development for paralysis across older patient populations.

## Data availability statement

The original contributions presented in the study are included in the article/supplementary material, further inquiries can be directed to the corresponding author.

## Ethics statement

The animal study was approved by the University of Utah Animal Care and Use Committee. The study was conducted in accordance with the local legislation and institutional requirements.

## Author contributions

NN: Investigation, Visualization, Writing – original draft, Writing – review & editing. MC: Formal Analysis, Investigation, Visualization, Writing – original draft, Writing – review & editing. PT: Conceptualization, Funding acquisition, Investigation, Methodology, Project administration, Resources, Supervision, Visualization, Writing – original draft, Writing – review & editing.
